# The Light Cupula Phenomenon: A Scoping Review

**DOI:** 10.3390/brainsci14010015

**Published:** 2023-12-23

**Authors:** Dong-Han Lee, Tae Hee Kim, Minho Jang, Chang-Hee Kim

**Affiliations:** Department of Otorhinolaryngology-Head and Neck Surgery, Research Institute of Medical Science, Konkuk University School of Medicine, Konkuk University Medical Center, Seoul 05030, Republic of Korea; 20200189@kuh.ac.kr (D.-H.L.); 20200135@kuh.ac.kr (T.H.K.); 20200123@kuh.ac.kr (M.J.)

**Keywords:** benign paroxysmal positional vertigo, dizziness, positional vertigo, light cupula

## Abstract

Direction-changing positional nystagmus (DCPN), which refers to the change in the direction of nystagmus with different head positions, is a well-known characteristic of horizontal semicircular canal BPPV. The supine head roll test is commonly used to diagnose horizontal canal BPPV. However, persistent geotropic DCPN observed during this test cannot be explained by the conventional explanations of canalolithiasis or cupulolithiasis. To account for this unique nystagmus, the concept of a “light cupula” has been recently introduced. In this review, we provide an overview of the historical background, clinical features and diagnostic methods, proposed mechanisms, and treatment strategies associated with the light cupula phenomenon based on the available literature to date.

## 1. Introduction

Benign paroxysmal positional vertigo (BPPV) is the most common cause of position-induced vertigo and nystagmus. Direction-changing positional nystagmus (DCPN), where the direction of induced nystagmus changes with different body positions, is a significant diagnostic feature observed in BPPV involving the horizontal semicircular canal (HSCC). The primary diagnostic tool employed for identifying HSCC BPPV is the supine head roll test. During this test, patients lie supine, with their head bent forward about 30 degrees, and then turn their head to either the right or left. This 30 degrees bending maximizes the effect of gravity on the otoliths within the HSCC by ensuring that the plane containing the HSCC is perpendicular to the ground. When conducting this test, two primary patterns of nystagmus may be manifested: (1) geotropic nystagmus, where nystagmus occurs with the head turned to either side, beating towards the ground, or (2) apogeotropic nystagmus, where nystagmus occurs in response to head movement, but beating in the opposite direction, away from the ground [1].

Based on current understanding, in HSCC canalolithiasis, which is described as the presence of free-floating otoconial debris in the HSCC, geotropic or apogeotropic nystagmus may occur depending on the location of the free-floating otoconial debris. In more detail, otoconial debris in the posterior arm of HSCC is thought to cause geotropic DCPN, while otoconial debris in the anterior arm of HSCC is believed to induce apogeotropic DCPN [2]. The nystagmus observed in HSCC canalolithiasis occurs after a short latency period. This is because, following a head movement, otolith debris is displaced within the HSCC due to gravity, causing endolymph to flow and subsequently resulting in cupula deflection. Afterwards, the endolymphatic flow stops, and nystagmus disappears within about 30 s to 1 min, which is called ‘transient’ geotropic (or apogeotropic) DCPN. On the other hand, in HSCC cupulolithiasis, a condition believed to be caused by the attachment of otoliths to the cupula, head turning leads to a more rapid onset (=shorter latency) of nystagmus due to the immediate bending of the cupula by gravity. Furthermore, in such cases, nystagmus presents as ‘persistent’ apogeotropic DCPN lasting for more than 1 to 2 min (Table 1).

However, the persistent geotropic DCPN induced during the supine head roll test is not well explained by the traditional mechanisms of canalolithiasis or cupulolithiasis. This unique nystagmus is believed to originate from a deflection of the HSCC cupula in the opposite direction to gravity. The ‘light cupula’ is a concept proposed to explain this type of persistent geotropic DCPN. In this review, we attempt to summarize the history, clinical features, diagnostic methods, putative mechanisms, and potential treatment of light cupula phenomenon by reviewing the literature to date.

## 2. Methods

This review includes studies based on a PubMed search on light cupula and related phenomena such as positional alcohol nystagmus through the period from the 1950s to 2023. A literature review was performed using PubMed database with keywords “light cupula”, “persistent geotropic direction changing positional nystagmus”, and “positional alcohol nystagmus”. We included reviews, animal studies, and all kinds of clinical studies. After reviewing titles and abstracts, we excluded papers that were less relevant or not written in English. Additionally, we examined relevant references cited in the reviewed literature.

## 3. History of the Concept of Light Cupula

**Figure 1:**
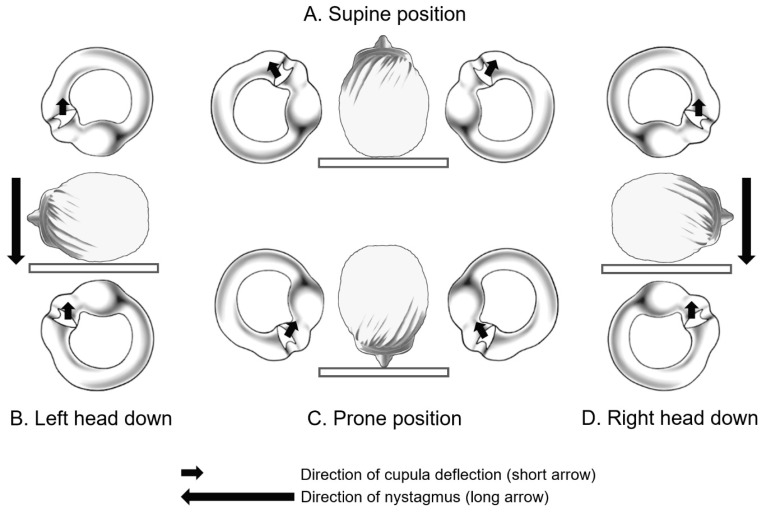
Persistent geotropic direction-changing positional nystagmus observed in positional alcohol nystagmus (PAN-I).

The origin of the concept of light cupula can be traced back to positional alcohol nystagmus (PAN). This phenomenon, characterized by persistent DCPN following alcohol consumption, was first described by Aschan and colleagues in 1956 [3]. Subsequent research by Money et al. in 1965 and 1974 provided additional explanations for the PAN phenomenon [4,5]. After alcohol consumption, ethanol with a specific gravity of 0.79 is thought to diffuse more rapidly from the bloodstream to the cupula than to the endolymph. This temporarily reduces the density of the cupula compared to that of the endolymph, causing persistent geotropic DCPN (PAN-I) in a “light cupula” state (see Figure 1). Conversely, if the blood alcohol concentration decreases over time, it can be hypothesized that the previously diffused alcohol has now been removed from the cupula more quickly than from the surrounding endolymph. This leads to the cupula becoming heavier than the surrounding endolymph, resulting in the occurrence of persistent apogeotropic DCPN (PAN-II).

**Figure 2:**
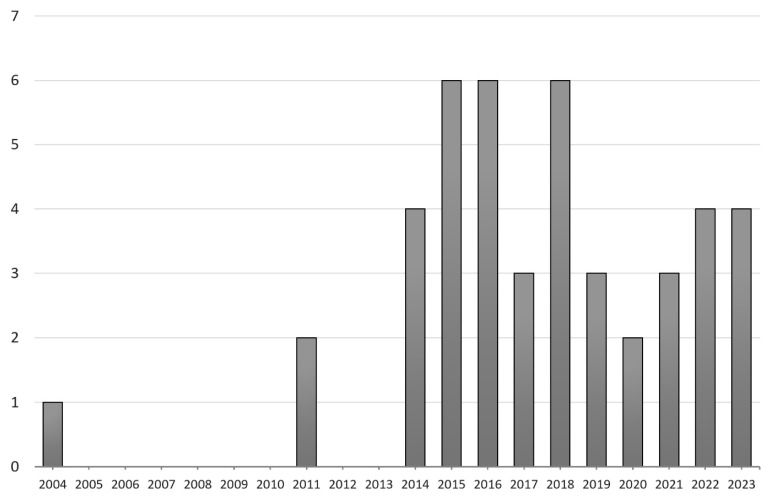
Number of English literature studies found by searching the keyword ‘light cupula’ in PubMed, by year (up to September 2023).

In 2002, Shigeno and colleagues proposed the concept of light cupula [6]. In 2004, Hiruma and colleagues observed a neutral point, where nystagmus disappeared in patients exhibiting persistent DCPN [7]. This neutral point occurred when patients turned their heads approximately 20–30 degrees from the supine position and rotated 180 degrees from that point. They reported that such a phenomenon could be well explained by assuming either light or heavy cupula. Subsequently, in 2014, Kim and colleagues, followed by Ichijo and colleagues in 2016, reported similar cases of both light and heavy cupula, which further increased the researchers’ interest in the concept of light cupula [1,8,9]. Recently in 2023, Peng and colleagues reported the clinical characteristics of a group of 189 patients with light cupula, which is the largest reported to date [10]. As of September 2023, a search on PubMed (https://pubmed.ncbi.nlm.nih.gov/ (accessed on 30 September 2023).) using the keyword “light cupula” yielded a total of 44 English-language publications, indicating a consistent and sustained interest among researchers over the past decade, particularly since 2014 (Figure 2).

## 4. Clinical Features and Diagnosis of Patients with Light Cupula

**Figure 3:**
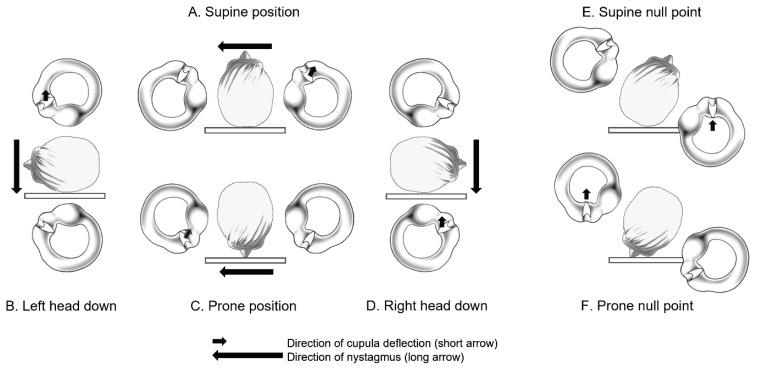
Persistent geotropic direction-changing positional nystagmus and null points observed in a patient with right-sided light cupula.

The typical symptom in patients with light cupula is vertigo related to changes in head position. As mentioned in the introduction, a characteristic feature of a light cupula patient is the presence of persistent geotropic DCPN during the supine head roll test. Assuming a lower density of cupula compared to the surrounding endolymph, this phenomenon of persistent geotropic DCPN can be well explained (Figure 3). The continuous bending of the cupula due to the difference in density between the cupula and endolymph can occur, even in the contrasting condition of a ‘heavy cupula’. The HSCC cupulolithiasis can also be understood as a condition where the otoconial debris are attached to the cupula, leading to a heavy cupula state. In this way, both light and heavy cupula can result in persistent DCPN (geotropic and apogeotropic, respectively), which is characterized by a shorter latency compared to transient DCPN and a longer duration of nystagmus lasting for more than 1–2 min.

**Figure 4:**
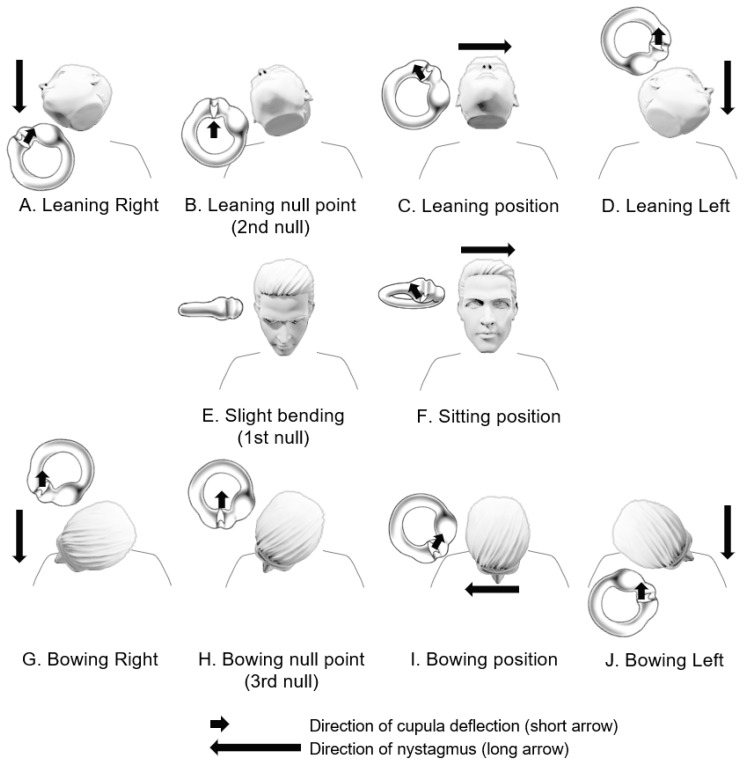
Bow and lean test and null points in a patient with right-sided light cupula.

Another common feature observed in both a light cupula and a heavy cupula is the presence of a specific plane known by various terms such as the null point, null plane, zero plane, neutral point, or neutral position. This plane is where vertigo and nystagmus disappear when head turning during the supine head roll test. To understand the null point, it is essential to have an anatomical understanding of the orientations of the HSCC and the axis of the cupula within the temporal bone. Although there have been historical misconceptions and revisions regarding this anatomical understanding in the literature until recent time [11,12], current knowledge suggests that the anterior portion of the HSCC is inclined upward by approximately 30 degrees relative to the horizontal plane. Additionally, the axis of the HSCC cupula is aligned anterior to posteriorly outward (laterally) by about 20–30 degrees relative to the sagittal plane, with the upper part of the cupula closely adhering to the canal wall (Figure 4) [8,13]. Therefore, when patients with a unilateral lesion rotate their head approximately 20–30 degrees toward the affected side during the supine head roll test, the axis of the HSCC cupula on the affected side aligns parallel to the gravitational vector. Consequently, the bending of the cupula disappears, leading to the cessation of vertigo and nystagmus at this point, and the direction of nystagmus reverses on either side (Figure 3). Therefore, to determine the affected side in patients with light cupula, it is important to know on which side the null point is observed.

In 2015, Kim and colleagues proposed that the same purpose could be achieved through the bow and lean test (Figure 4) [8]. In the upright sitting position, because the HSCCs are inclined forward by approximately 30 degrees, in patients with a light cupula, the affected side cupula may bend in the ampullofugal direction, inducing nystagmus beating toward the opposite side of the lesion (Figure 4F). At this point, if the head is slightly bowed, the HSCC aligns parallel to the ground, and the first null point can be observed (Figure 4E). In terms of head position, leaning the head back while sitting upright is similar to bending the head 30 degrees forward while lying down, which is the typical supine head roll test position (Figure 4C). Therefore, in the head-leaning position while sitting upright, turning the head from side to side can lead to persistent geotropic DCPN in light cupula patients and persistent apogeotropic DCPN in heavy cupula patients (Figure 4A,D). Furthermore, when the head is slightly turned towards the affected side, a second null point appears (Figure 4B). Similarly, when the head is bent forward (bowing) in a seated position, and the patients turn the head from side to side, we can observe persistent geotropic DCPN in light cupula patients and persistent apogeotropic DCPN in heavy cupula patients (Figure 4G,I,J). Also, in the bowing position with a slight head turning towards the affected side, a third null point appears (Figure 4H).

In this manner, assuming either light or heavy cupula, theoretically, persistent DCPN can occur in all positions except the null points. For example, nystagmus can occur even in an upright sitting position or a supine position, and this can be taken into consideration during diagnosis and in determining the affected side. Figure 3 and Figure 4 illustrate that in a right-sided light cupula patient with a supine position, left-beating nystagmus is observed (Figure 3A), whereas when the head is bent forward (bowing position) in a sitting position, right-beating nystagmus is observed (Figure 4I). Furthermore, during the supine head roll test, the intensity of nystagmus is stronger when turning the head toward the affected ear (Figure 3B,D). This is because head turning toward the affected side causes ampullopetal deviation of the cupula in the affected ear, resulting in an excitatory response, leading to a stronger nystagmus intensity, whereas head turning toward the healthy side causes ampullofugal deviation of the cupula in the affected ear, resulting in an inhibitory response, leading to a weaker nystagmus intensity, following Ewald’s second law.

Although the PAN phenomenon provided clues to the origin of the light cupula concept, from a null point perspective, it is different from light cupula. Theoretically, in the case of unilateral light or heavy cupula, the HSCC cupula on the unaffected side has a density equal to that of the surrounding endolymph, making it unaffected by gravity. However, in PAN, the cupula density on both sides changes simultaneously, subjecting it to the influence of gravity. Therefore, it is estimated that the gravity effects on both sides cancel each other out when lying in a supine or prone position, resulting in the absence of nystagmus, as depicted in Figure 1.

When considering the characteristics discussed above, the diagnosis of light cupula of the HSCC can be made based on the following four criteria. (1) There is continuous positional vertigo triggered by specific head positions, and during the supine head roll test, there is persistent geotropic DCPN lasting for more than one minute. This nystagmus has minimal latency, and does not exhibit fatigability. (2) The null point can be identified. (3) The affected side corresponds to the side where the null point is observed. (4) There are no central nervous system abnormalities [1,9,14,15,16].

According to a study by Kim in 2014, the incidence of such light cupula was reported to be 4.9% among a total of 388 patients with BPPV over a period of approximately 1.5 years. Among the patients with DCPN, the incidence was 9.4%, and among the patients with geotropic DCPN, it was 14.2% [1]. Furthermore, the natural course of light cupula is known to require a longer period until recovery compared to the typical course of HSCC BPPV. According to a study by Kim and colleagues in 2018 [17], which tracked 65 patients with persistent geotropic DCPN, there was no statistically significant difference in the time to resolution of nystagmus or symptom improvement between the treatment group (*n* = 35) and the non-treatment group (*n* = 30). The time required from diagnosis to resolution of nystagmus was approximately 8 days or more, which is longer than the previously reported natural course of 4.7 ± 3.9 days (canalolithiasis) and 4.4 ± 5.0 days (cupulolithiasis) for patients with HSCC BPPV [18]. Additionally, reports on the incidence of light cupula have generally indicated a higher prevalence among women as compared to men [10,16,19]. BPPV is also generally reported to have a higher incidence among females compared to males [20]. It is estimated that several factors such as hormones, age, osteoporosis, and head trauma may influence the incidence of BPPV [21]. Although we explore the underlying mechanism of light cupula in the following section, if light cupula phenomenon arises from a different mechanism from traditional BPPV, it raises the need for additional research to provide a satisfactory explanation for the higher prevalence among women.

## 5. Mechanisms

While the mechanism of light cupula remains unclear, it is generally believed to be related to a problem within the vestibular system [22,23]. Several mechanisms have been proposed, including the following, listed in the sub-sections below.

### 5.1. Lighter Cupula Theory

The lighter cupula theory suggests that light cupula is caused by a decrease in the density of the cupula within the semicircular canal. The previously discussed positional alcohol nystagmus (PAN-I) is believed to be related to this mechanism [4]. For example, in the case of right-sided light cupula, when the right ear is positioned downward, the cupula’s density in the right HSCC is lower than that of the surrounding endolymph. This difference in density causes buoyancy, which bends the stereocilia of the hair cells towards the kinocilium, leading to an excitation of the right HSCC. On the other hand, when the left ear is positioned downward, buoyancy causes the stereocilia of the right HSCC to deflect away from the kinocilium, thereby inhibiting the right HSCC. In both positions, the direction of nystagmus is towards the ground (Figure 3). The lighter cupula theory received support from subsequent research, including a study by Money and colleagues in 1974, which observed that heavy water (deuterium oxide) intake could induce a heavy cupula condition [5]. In 1989, Shigeno and colleagues conducted animal experiments using cats and rabbits, confirming that persistent geotropic or apogeotropic DCPN could be induced by injecting solutions with densities lower or higher than endolymph into the middle ear cavity. Histological analysis revealed the accumulation of eosinophilic materials near the round window membrane and the cupula, suggesting that the injected material probably passed through the round window membrane from the middle ear to the endolymphatic system of the inner ear and accumulated in the cupula [24].

In 1965, Money and colleagues observed whether PAN appeared after consuming alcohol in cats with certain semicircular canals removed or inhibited [4]. When the cat’s head was held in either an upward or downward position, alcohol affected both semicircular canals simultaneously, resulting in a cancellation of inhibition or excitation of both semicircular canals, and consequently, there was no occurrence of PAN. However, when one semicircular canal was removed or inhibited, it resulted in horizontal PAN. In cases where both HSCCs were inhibited, no horizontal PAN was observed in any position, but vertical or torsional components of PAN were still present. In a study by Tomanovic and Bergenius in 2011 [12], they observed that patients with bilateral labyrinthectomy did not exhibit PAN, whereas patients with unilateral labyrinthectomy showed PAN corresponding to a unilateral light cupula. Meanwhile, sulfated proteoglycan found in the endolymphatic sac was reported to be synthesized in the cupula and secreted into the endolymph. In 2006, Bergenius and Tomanovic suggested that changes in the homeostasis of these large molecules would change the relative density of the cupula to the endolymph [11]. In 2015, Seo and colleagues reported a case of a patient with sudden sensorineural hearing loss who had incurable, persistent geotropic DCPN for more than 6 months and was treated with HSCC plugging surgery. They suggested that the cause of the intractable light cupula might be related to an irreversible morphological change in the cupula (enlarged cupula) In other words, it was assumed that the density of the cupula decreased due to the enlargement of the cupula while the mass of the cupula remained constant [25].

### 5.2. Heavier Endolymph Theory

This theory assumes that the density of the endolymph may increase due to acute inner ear injury caused by labyrinth hemorrhage, inner ear hypoperfusion, or inflammatory response [1]. In 2011, Hiruma and colleagues reported cases of patients who developed light cupula syndrome after stellate ganglion block [14]. They proposed that increased blood flow in the vertebral artery following stellate ganglion block might lead to inner ear hypoperfusion, resulting in changes in the density or viscosity of the inner ear. In 2014, Kim and colleagues observed that some patients with sudden hearing loss and vertigo showed persistent geotropic DCPN, and suggested that damage to the blood–labyrinthine barrier might result in the leakage of plasma proteins into the inner ear fluid, potentially increasing the specific gravity of the endolymph [26,27,28]. This hypothesis has been supported by reports of cases in which persistent geotropic or apogeotropic DCPN was observed alternating over time, which was hypothesized to be due to overcompensation of endolymphatic homeostasis [29].

There are two main criticisms regarding the ‘heavier endolymph’ theory: (1) the rapid onset of symptoms and short clinical course cannot be reasonably explained by changes in the specific gravity of the endolymph, and (2) in most cases of light cupula, HSCC is the only affected semicircular canal. However, when a light cupula is associated with sudden hearing loss [26] or meningitis [30], vertigo is usually reported to occur hours to days after the onset of hearing loss or meningitis symptoms. It is presumed that this is because it takes time for the density of vestibular endolymph to increase. In addition, it was reported that it takes 8 days to 2 weeks for vertigo or nystagmus to disappear in patients with light cupula [1,17], which is much longer than the natural course of untreated HSCC canalolithiasis or cupulolithiasis [18]. Regarding the second criticism, since endolymphatic fluid circulates within inner ear organs connected by endolymphatic membranes, it is a reasonable criticism that if ‘heavier endolymph’ is the underlying mechanism, this phenomenon should also occur in the three semicircular canals and otolith organs. Although a recent case was reported in which light cupula involved all three semicircular canals on the affected side [31], all previous reports mainly focused on cases involving HSCCs. There has been speculation that the predominance of horizontal nystagmus in the light cupula may be because the vertical and torsional components of the nystagmus are combined or canceled by excitation and inhibition of the vertical semicircular canals [1,31]. Meanwhile, the exact arrangement of the cupula within the vertical semicircular canal with respect to gravity is not yet clearly known, so further research is needed. In an animal study by Money and colleagues in 1965 [4], when the bilateral HSCCs were inactivated, the horizontal component of PAN was not observed, but the vertical and torsional components were still present. Based on these findings, in 2004, Hiruma and Numata speculated that the vertical and torsional components of nystagmus in light cupula may be hidden [7].

### 5.3. Light Debris Theory

The light debris theory suggests that particles lighter than the endolymph attach to the cupula of the HSCC, making the cupula lighter [9,11,22,32,33]. This hypothesis is supported by the fact that, in the majority of light cupula cases, the horizontal component of DCPN is the most prominent. Furthermore, the sudden onset of positional vertigo, unilateral involvement, and the tendency for dizziness and nystagmus to gradually improve over time are consistent with this theory. Based on this theory, it is possible to explain that clinical characteristics may differ depending on whether the light debris is attached to the utricular side or the canal side of the cupula. Several candidate materials for such light debris have been proposed, including (1) inflammatory cells that have degenerated, swollen, and become lighter within the endolymph, or (2) substances possibly formed through a chemical reaction from otolith debris [14,33,34]. In some cases, the conversion from persistent apogeotropic to geotropic DCPN in a patient could be explained by the hypothesis that attached otoconial particles expand and become lighter. In 2016, Ichijo demonstrated that the angle of null point is greater in light cupulas compared to heavy cupulas, suggesting that light debris may be more commonly attached to the canal side of the HSCC cupula than the utricular side [9]. However, this hypothesis is also being challenged as attempts to treat ‘light debris’ by liberating it from the cupula have not been successful so far [17,35], and there have been no confirmed reports of light debris to date.

### 5.4. Utricular Macula Theory

As another theory, in 2004, Hiruma and colleagues observed persistent geotropic DCPN in patients with peripheral auditory–vestibular disorders, and suspected that dysfunction of the utricular macula could potentially be the pathophysiological basis for this nystagmus [7]. However, there is a counter-argument that dysfunction of the utricular macula alone is insufficient to explain such nystagmus [11]. In 2023, Ichijo and colleagues assumed that pathologic debris would be present in the utricle immediately after successfully performing canalith repositioning procedures in 12 patients with horizontal and posterior semicircular canal BPPV. They tilted the head of a patient in various directions to allow the pathologic debris to directly stimulate the utricle. However, they observed neither nystagmus nor dizziness in all 12 patients, and concluded that nystagmus would not be caused by stimulation of the otolith organ alone [36].

### 5.5. Density Difference between Perilymph and Endolymph

In 2019, Kim and colleagues proposed that density differences between the perilymph and endolymph may influence the phenomenon of light cupula [37]. If the density of the perilymph increases and becomes higher than that of the endolymph, the semicircular canal filled with endolymph will experience greater buoyancy within the surrounding perilymph under gravity. Because the endolymphatic membrane is thin and has deformable characteristics, the deformation of the lymphatic duct due to the physical force of buoyancy can displace endolymph, leading to the deflection of the cupula and resulting in characteristic persistent geotropic DCPN.

In summary, the first three theories assume a situation where the cupula is relatively light compared to the surrounding endolymph, explaining persistent geotropic DCPN. However, the latter two theories propose that persistent geotropic DCPN may arise from mechanisms other than a lighter cupula. In other words, the concept of a light cupula is just one of several theories explaining persistent geotropic DCPN, and there is still controversy regarding the use of “light cupula” itself as a disease name. Persistent geotropic DCPN is a term primarily based on observational findings, while “light cupula” is a term indicating one of its pathophysiological mechanisms. It is important to note that these two terms may not always align. Additionally, the mechanisms proposed above may not be mutually exclusive. Parts of each mechanism may contribute to persistent geotropic DCPN, and there might be additional, undiscovered mechanisms. Therefore, further research seems necessary to explore these aspects.

## 6. Etiologies and Differential Diagnosis

### 6.1. Vestibular Migraine and Light Cupula

Although the mechanism of light cupula is still debated, it has been reported that the most common nystagmus pattern observed during the acute phase of vestibular migraine is persistent geotropic DCPN. In 2004, Brevern and colleagues observed persistent geotropic DCPN, persistent apogeotropic DCPN, and transient pure torsional nystagmus in individual patients with vestibular migraine during acute attacks [38]. In 2010, Polensek and Tusa reported that persistent DCPN was most commonly observed positional nystagmus in patients with acute vestibular migraine [39]. Although it has not been clearly reported whether this central positional nystagmus was geotropic or apogeotropic, it was generally slow, persistent, had no latency, was only observed during the headache attack, and could not be explained as any type of BPPV. Furthermore, vestibular migraine could not explain the presence of the null points well. In 2014, Lechner and colleagues observed persistent geotropic DCPN in 5 out of 13 patients with acute vestibular migraine [40]. This nystagmus, characterized by its bilateral symmetry and slow velocity during the supine head roll test, was distinct from the rapid crescendo–decrescendo pattern typically seen in HSCC canalolithiasis. In 2014, Tomanovic and Bergenius followed 20 patients with sudden vestibular dysfunction and persistent geotropic DCPN, and found that 8 of the 20 (40%) had a history of migraine and met the diagnostic criteria for vestibular migraine [35]. Notably, 65% of these patients were women. In 2012, Radtke and colleagues found positional nystagmus in 28% of patients with vestibular migraine and suggested that these changes may be related to vascular spasm in the inner ear or brainstem [41]. The high proportion of patients with vestibular migraine and the absence of central nervous system abnormalities in their study suggest that vestibular migraine is associated with peripheral vestibular dysfunction. In 2004, Brevern and colleagues proposed dysfunction of inhibitory GABAergic connections from the vestibulocerebellum to the vestibular nuclei as a cause of persistent geotropic DCPN in vestibular migraine patients [38]. In addition, inner ear damage due to vasospasm of the labyrinthine arteries or trigeminovascular activation is one of the candidate mechanisms that can explain the persistent geotropic DCPN observed in vestibular migraine [42]. Various studies are strengthening the pathophysiological connection between migraine and positional vertigo and nystagmus [43].

### 6.2. Central Lesions and Light Cupula

In some patients with central lesions, persistent geotropic DCPN can be observed. In 2014, Lee and colleagues reported two cases initially diagnosed with HSCC canalolithiasis, that were eventually found to have central lesions, specifically cerebellar glioma and lateral medullary infarct, respectively [44]. In 2014, Yang and Oh reported persistent geotropic DCPN in a patient with human immunodeficiency virus encephalopathy [45]. They noted that when turning the head to the left, the nystagmus was stronger than to the right, there was no spontaneous nystagmus, and there was no nystagmus during the bow and lean test. In 2017, Choi and colleagues reported a case of light cupula due to meningitis [30]. They speculated that the increased protein concentration in cerebrospinal fluid might have increased the protein content in endolymph, leading to an increased density of the endolymph. In 2018, Choi and colleagues reported that 12% of patients with persistent geotropic DCPN had central lesions [46]. In 2015, Imai and colleagues demonstrated that the time constant of the slow phase velocity of persistent geotropic DCPN is similar to that of persistent apogeotropic DCPN [22]. They suggested that since the pathophysiological basis of persistent apogeotropic DCPN is considered to be heavy cupula, persistent geotropic DCPN may be conversely induced by light cupula of peripheral origin. Considering the axis of the HSCC cupula, the presence of null points was considered an important clinical characteristic of cupulopathy [8]. In 2018, Choi and colleagues reported that null points could also be observed in central persistent geotropic DCPN, but they might not always be observed on the lesion side [46]. They also reported that the stronger and more asymmetric the nystagmus in persistent geotropic DCPN, the more likely that it indicates a peripheral origin. If null points are confirmed on the lesion side, and there are no other abnormalities on neurological examination, it is reasonable to consider peripheral cupulopathy as the cause of persistent geotropic DCPN [1,7,8,14,33].

### 6.3. Other Inner Ear Disorders and Light Cupula

In 1957, Aschan and Stahle described three out of 21 patients with Meniere’s disease who exhibited persistent geotropic DCPN during the period of acute vertigo, and suggested that the mechanism of geotropic DCPN in Meniere’s disease was similar to that in PAN [47]. Although this study did not confirm null points, it can be assumed that null points might have been identified on the contralesional side based on the nystagmus records of all three patients. Cases of persistent geotropic DCPN have also been reported in patients with sudden sensorineural hearing loss [11,14,25,26,27,28], acute otitis media with serous labyrinthitis [48], and Ramsay Hunt syndrome [49]. Compared to general light cupula patients, the null points may not be identified in patients with light cupula accompanied by ipsilateral inner ear disorders, and a transition from geotropic to apogeotropic nystagmus may be observed more frequently [29,48]. Several reports have documented cases of transition between persistent geotropic DCPN and apogeotropic DCPN, which can provide clues regarding the mechanism of light cupula. In 2015, Shin and colleagues reported a case in which a patient with sudden sensorineural hearing loss and persistent geotropic DCPN transitioned to persistent apogeotropic DCPN. They suggested that initially light cupula might have occurred due to an increase in endolymphatic density, which was then converted to heavy cupula by attachment of displaced otoconial debris to the cupula [29]. Similarly, in cases of conversion from apogeotropic to geotropic DCPN, it is possible that the otoconial debris attached to the cupula become swollen, reducing its density and resulting in a change in the direction of nystagmus. It is also speculated that this conversion phenomenon may be due to overcompensation of the endolymph homeostasis [29].

## 7. Treatments

Although effective treatments for persistent geotropic DCPN have not yet been established, several treatment methods have been attempted, and the results of these treatments may conversely provide clues to the underlying mechanisms of light cupula. In one study, the barbecue roll maneuver, a traditional canalith repositioning technique for transient geotropic DCPN, was performed to treat persistent geotropic DCPN, but it was reported to be ineffective [16]. Meanwhile, the immediate success rate of the Gufoni maneuver in patients with apogeotropic DCPN was reported to be approximately 50% [2]. Although this study did not analyze the cases of apogeotropic DCPN by dividing them into ‘transient’ (HSCC canalolithiasis of anterior arm) and ‘persistent’ (HSCC cupulolithiasis) subtypes based on the duration of nystagmus, it is presumed that many patients with so-called ‘heavy cupula’ were included, and they responded to the appropriate repositioning maneuver. This result aligns with the previous understanding that otoconial debris attached to the cupula primarily contributes to the mechanism of a heavy cupula. In contrast, the reported success rate of repositioning maneuvers in patients with light cupula showing persistent geotropic DCPN is 0%, which suggests that the cause of nystagmus is the deflection of the cupula itself by other mechanism rather than light debris attached to the cupula. In 2018, a study attempted a new repositioning maneuver that modified the existing maneuver for cupulolithiasis [50], to treat the light cupula [17]. This new repositioning maneuver was designed to use mastoid oscillation to dislodge light debris from the cupula, allowing it to float in the opposite direction of gravity and move into the utricle. However, this approach was not found to be effective in treating persistent geotropic DCPN. These observations suggest that factors other than light debris, such as a lighter cupula or heavy endolymph, may be more appropriate mechanisms to explain persistent geotropic DCPN.

In 2016, Cha and colleagues reported a patient with light cupula whose positional vertigo disappeared immediately after transcutaneous vagus nerve stimulation, but the mechanism was not clearly explained, and nystagmus reappeared later [51]. In most patients with light cupula, vertigo or positional nystagmus resolves spontaneously within two weeks, but in patients with concomitant sudden sensorineural hearing loss, positional vertigo can persist for several months or longer [25,28]. In some cases, surgery such as semicircular canal plugging may be required to treat intractable positional nystagmus [25].

Intratympanic steroid injections have been used in the treatment of certain inner ear disorder conditions such as sudden sensorineural hearing loss, tinnitus, and Meniere’s disease. They are known to suppress inner ear inflammation, improve inner ear blood circulation, and help maintain lymphatic fluid homeostasis. In 2018, Park and colleagues reported that intratympanic steroid injections may somewhat alleviate dizziness symptoms in patients with light cupula, but did not report a statistically significant effect [15].

The natural course of light cupula is known to be rather long, and it may be accompanied by other inner ear diseases. It is best to be diagnosed as early as possible to avoid unnecessary repositioning maneuvers. When patients have a previous history of Meniere’s disease, vestibular migraine, sudden sensorineural hearing loss, or central nervous system disorders, the possibility that light cupula may manifest as part of the clinical presentation of these conditions should be considered. In such cases, both symptom management and treatment for the underlying condition should be provided.

## 8. Conclusions

According to the clinical practice guidelines for BPPV by the American Academy of Otolaryngology-Head and Neck Surgery [20], lastly updated in 2017, the definition of BPPV is “A disorder of the inner ear characterized by repeated episodes of positional vertigo.” However, the guideline does not yet currently include information about the light cupula phenomenon. Since the concept of light cupula became widely known after the mid-2010s, it has become newly applicable to some patients who did not fit the traditional diagnostic criteria of BPPV or did not respond well to repositioning maneuvers. Furthermore, based on the concepts of light and heavy cupula discussed so far, it is possible that some patients previously diagnosed with HSCC cupulolithiasis may have underlying mechanisms other than otoconial debris attached to the cupula. This may explain why they do not respond as well to canalith repositioning procedures compared to other subtypes of BPPV. The concept of light cupula has contributed to a better understanding of previously confusing phenomena and diagnoses. It now enables more accurate identification of patients with a light cupula, potentially sparing them from unnecessary repositioning maneuvers or additional tests. However, the exact cause and underlying mechanisms of light cupula are still not fully understood. Therefore, further research on the anatomical and molecular mechanisms of light cupula is expected to enhance our understanding of DCPN and enable the development of effective treatment strategies in the future.

## Figures and Tables

**Table 1:** Differential diagnosis of horizontal semicircular canal disorders presenting with direction-changing positional nystagmus.

	HC Canalolithiasis (Posterior Arm)	HC Canalolithiasis (Anterior Arm)	HC Cupulolithiasis (Heavy Cupula)	Light Cupula
**DCPN**	Geotropic	Apogeotropic	Apogeotropic	Geotropic
**Duration**	Transient	Transient	Persistent	Persistent
**Latency**	+	+	-	-
**Fatigability**	+	+	-	-
**Null plane**	-	-	+	+

HC: horizontal canal, DCPN: direction-changing positional nystagmus.

## Data Availability

This study does not contain new data.
